# The tooth transformer® revolution: autologous dentin biomaterials and platelet concentrates in oral regeneration. A parallel narrative systematic review

**DOI:** 10.3389/fdmed.2025.1750541

**Published:** 2026-01-16

**Authors:** Gianna Dipalma, Grazia Marinelli, Laura Ferrante, Irma Trilli, Sharon Di Serio, Paola Bassi, Francesco Inchingolo, Andrea Palermo, Angelo Michele Inchingolo, Alessio Danilo Inchingolo

**Affiliations:** 1 Interdisciplinary Department of Medicine, University of Bari “Aldo Moro”, Bari, Italy; 2 Department of Biomedical, Surgical and Dental Sciences, Milan University, Milan, Italy; 3 Department of Experimental Medicine, University of Salento, Lecce, Italy

**Keywords:** alveolar ridge augmentation, autologous dentin, autologous platelet concentrates, bone graft materials, bone regeneration, concentrated growth factors, PRF, tooth transformer®

## Abstract

**Aim:**

Bone regeneration in oral and maxillofacial surgery remains a clinical challenge, particularly in cases of severe alveolar atrophy or large post-pathological bone defects. Autologous biomaterials including dentin grafts processed with the Tooth Transformer® device and platelet concentrates such as Concentrated Growth Factors (CGF), PRF, i-PRF, and TPRF—have gained attention due to their biocompatibility and regenerative potential. However, no direct head-to-head clinical studies comparing these autologous approaches are currently available.

**Materials and methods:**

This review was conducted according to PRISMA 2020 guidelines. Literature searches were performed in PubMed, Scopus and Web of Science for clinical studies published in English between January 2010 and December 2025. Eligible designs included randomized trials, prospective/retrospective studies, and case series with ≥5 patients. Owing to the paucity of CGF-only studies, the comparison was broadened to include platelet concentrates (CGF/PRF/L-PRF/i-PRF/TPRF). Outcomes included bone volume gain, radiographic density, histologic new bone formation, healing time, implant survival, and postoperative complications.

**Results:**

The search yielded 1,004 records; 10 studies met the inclusion criteria. Autologous dentin grafts demonstrated favorable volumetric stability and bone density compatible with mature bone, while platelet concentrates consistently improved early healing, angiogenesis, and trabecular organization—particularly in contained defects. Substantial methodological heterogeneity and the absence of direct comparative trials precluded quantitative synthesis.

**Conclusions:**

Autologous dentin grafts and platelet concentrates appear to play complementary roles: the former provides a structural scaffold for three-dimensional augmentation, whereas the latter enhances biological healing and tissue maturation. Combined protocols show promise but require validation through well-designed, multicenter randomized controlled trials.

## 1 Introduction

Bone regeneration in oral and maxillofacial surgery represents one of the most challenging frontiers in modern implantology and oral rehabilitation (1–6). Severe bone atrophy or large defects of the alveolar ridges, often the result of chronic inflammation, odontogenic cysts, traumas, periodontal disease, or prolonged edentulism, can compromise the functional and aesthetic outcome of implant-supported prosthetic rehabilitation (7–13). The re-establishment of adequate bone volume and quality is essential for achieving long- term osseointegration and biomechanical stability of dental implants. However, the biological complexity of bone tissue and the multifactorial causes of its resorption make regeneration a difficult and often unpredictable process (14–21).

Over the last three decades, regenerative medicine and biomaterial science have significantly advanced the understanding of bone healing with the neoapposition of new autologous bone and tissue engineering (22–29). The principles of osteogenesis, osteoconduction, and osteoinduction are at the core of current regenerative strategies, and the selection of appropriate grafting material plays a critical role in determining clinical success (30–36). Despite these advances, bone regeneration still poses considerable biological and technical challenges, especially in anatomically demanding areas such as the posterior maxilla or in cases with vertical and horizontal bone deficiency (37–41).

Traditionally, bone augmentation has been performed using autologous bone grafts, considered the gold standard for decades due to their intrinsic osteogenic, osteoconductive, and osteoinductive properties (42–49). Nevertheless, autologous grafts require intraoral or extraoral harvesting procedures, which increase surgical morbidity, operative time, postoperative discomfort, and the risk of donor site complications. Furthermore, autologous bones are prone to resorption during remodeling, which can compromise volume stability in the long term (50–56). Alternative materials such as allografts, xenografts, and synthetic bone substitutes have been introduced to overcome these drawbacks. These substitutes act primarily as osteoconductive scaffolds that provide structural support but are not completely replaced by newly formed bone; as a result, they can prolong remodeling and delay the maturation of neoformed bone (57–65). Moreover, they typically lack viable cells, which may contribute to slower or incomplete replacement. Histologic studies have shown that even after 24–30 months, newly formed bone in grafted sites with xenografts or synthetic materials typically does not exceed 40%–50% of the total regenerated tissue (66–75). The remaining portion is composed of residual biomaterial and connective tissue, which may affect the long-term mechanical behavior of the implant–bone interface (76–86). Additionally, issues such as immunogenic response, slow resorption kinetics, cost, and inconsistent biological integration continue to limit their predictability (87–89). Given these limitations, research in regenerative dentistry has increasingly focused on autologous biomaterials derived from the patient's own tissues or blood (90–96). These biologically compatible materials abolish immunological reactions, eliminate the risk of disease transmission, and actively promote the natural healing cascade (97–99). Among these, two innovative approaches, autologous dentin grafts and Concentrated Growth Factors (CGF) (MEDIFUGE 200® SILFRADENT, Santa Sofia, Italy), have emerged as promising alternatives to conventional grafts, thanks to distinct biological mechanisms that accelerate bone regeneration (100–108).

### 1.1 Autologous dentin as a biomaterial

Autologous dentin grafts are obtained by recycling a patient's extracted teeth into a biocompatible bone substitute (109–117). This concept relies on the chemical and structural similarity between dentin and bone tissue (118–121). Dentin consists of approximately 70% inorganic phase (mainly hydroxyapatite), 20% organic matrix (predominantly type I collagen), and 10% water, closely mirroring the composition of cortical bone (122–125). The organic phase also contains a rich array of bioactive non-collagenous proteins such as bone morphogenetic proteins (BMPs), transforming growth factor beta (TGF-β), insulin-like growth factors (IGF) (126), osteopontin, and dentin sialophosphoprotein, all of which play crucial roles in osteoblast differentiation and bone remodeling (127–136). When appropriately processed, dentin exhibits both osteoconductive and osteoinductive properties, providing not only a physical scaffold for new bone formation but also molecular cues that activate cellular migration and differentiation, leading to the rapid replacement of the graft material with newly formed bone, typically within 4–12 months (137–143).

The introduction of the Tooth Transformer® device *(*TT Tooth Transformer s.r.l., Milan, Italy*)* has standardized and simplified the clinical use of dentin as a grafting material (144–154). This medical device performs an automated process of cleaning, decontamination, demineralization, and sterilization, resulting in a ready-to- use granular biomaterial within 30–40 min after tooth extraction (155–159). The demineralization step exposes and activates the bioactive molecules within the dentin matrix, enhancing its regenerative potential (122, 160–162). The granules, typically ranging from 500 to 1,000 µm, can be easily adapted to various surgical sites, providing excellent handling characteristics and stability (163–168). Several preclinical and clinical studies have reported encouraging results with autologous dentin grafts in procedures such as alveolar ridge preservation, sinus floor elevation, lateral and vertical ridge augmentation, and treatment of cystic bone defects (169–174). Histological analyses have demonstrated external evidence has reported substantial new bone formation, although such values were not in the studies included in this systematic (175, 176). Moreover, the absence of donor site morbidity and the autologous nature of the graft make it a highly attractive option from both biological and ethical standpoints (177–188).

### 1.2 Concentrated growth factors (CGF)

**Figure 1:**
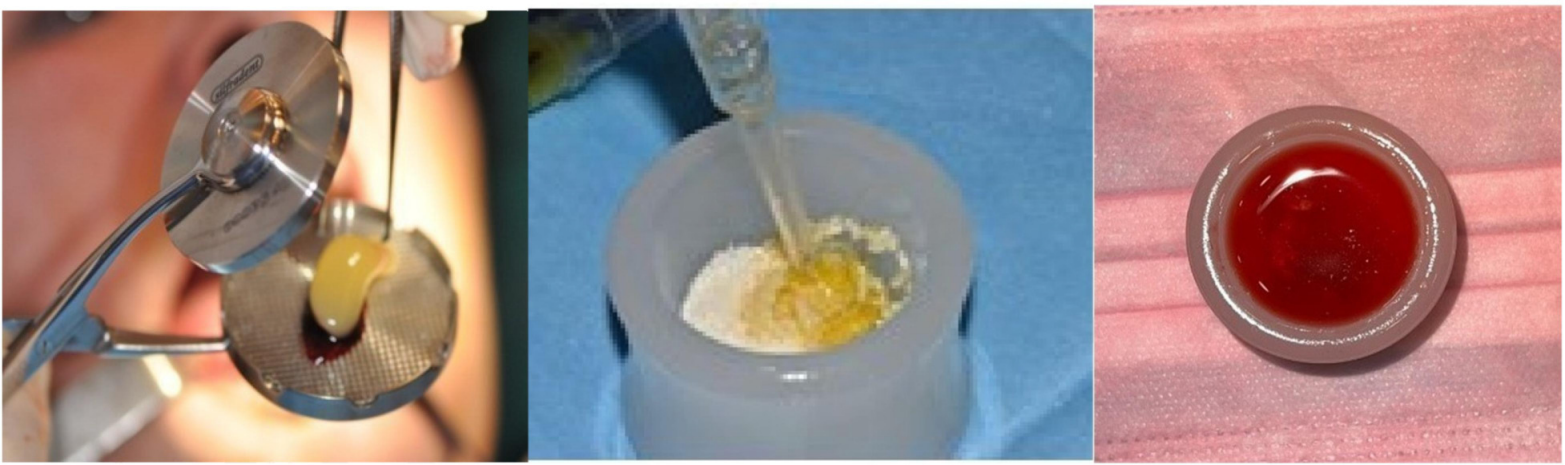
“Sticky bone” formed by combining CGF with particulate biomaterial, promoting tissue remodeling and bone regeneration. Original illustration created by the authors.

In parallel with developments in solid biomaterials, research in autologous blood derivatives has led to the emergence of platelet concentrates such as Platelet-Rich Plasma (PRP), Platelet-Rich Fibrin (PRF), and more recently, CGF (189–205). These preparations aim to exploit the regenerative potential of platelets and growth factors naturally present in the blood to enhance tissue repair and angiogenesis (206–209). CGF, first described by Sacco in 2006, represents the most advanced generation of platelet concentrates (99, 210–212). They are produced by centrifuging venous blood using a specific centrifuge, following a precise program that allows the differential separation of blood components without the need for anticoagulants or chemical additives (122, 213–218). The resulting fibrin matrix is denser and more elastic than those obtained with PRP or PRF, and it contains a higher concentration of growth factors, including platelet-derived growth factor (PDGF), vascular endothelial growth factor (VEGF), TGF-β1 and insulin-like growth factor IGF (219–222). This three- dimensional fibrin scaffold provides a sustained release of cytokines and supports cell proliferation, differentiation, and vascular ingrowth. In bone regeneration, CGF has been shown to accelerate osteoblast differentiation, enhance angiogenesis, and reduce inflammation. The presence of leukocytes and CD34 + stem cells further contributes to tissue remodeling and the stability of the wound through the formation of newly regenerated bone. Clinically, CGF can be used alone as a membrane or clot for minor defects, or in combination with particulate biomaterials to form the so-called “sticky bone”, a cohesive and moldable graft (223, 224). (Figure 1).

When combined with dentin particulate obtained through the Tooth Transformer, the granules become stabilized into a compact, easy-to-handle mass, which is highly advantageous and often essential for bone grafting procedures in the oral cavity (225, 226).

However, while the synergistic effect of CGF with other materials has been investigated, the independent regenerative potential of CGF, when used as the sole autologous component, remains a critical area of research, especially when compared to structural biomaterials such as autologous dentin (227–229).

Although both Tooth Transformer® dentin grafts and CGF are autologous materials that have demonstrated remarkable biocompatibility and regenerative capacity, they differ fundamentally in structure, biological function, and clinical handling. Dentin, once processed, provides a solid, mineralized scaffold capable of maintaining space and supporting mechanical load, whereas CGF provides a biological matrix enriched in signaling molecules that guide tissue repair through angiogenesis and cell activation rather than structural support. These differences raise important questions regarding their relative efficacy and predictability in bone regeneration (230–234). While autologous dentin grafts may provide greater volumetric stability and long-term integration, CGF may offer superior early-stage healing, enhanced vascularization, and improved soft-tissue management. Moreover, the growing clinical interest in both materials, along with the proliferation of case reports and small trials, has led to a fragmented body of evidence without clear consensus. To date, most studies have evaluated either the clinical success of Tooth Transformer® grafts or the biological potential of CGF independently, but few comparative analyses have systematically examined outcomes across the two approaches. Furthermore, the heterogeneity of study designs, follow-up durations, and evaluation methods complicates direct interpretation. Thus, a comprehensive systematic review is warranted to synthesize existing evidence, identify methodological gaps, and guide clinical decision-making regarding which autologous strategy may offer the most predictable outcomes in different clinical scenarios (235–237).

**Figure 2:**
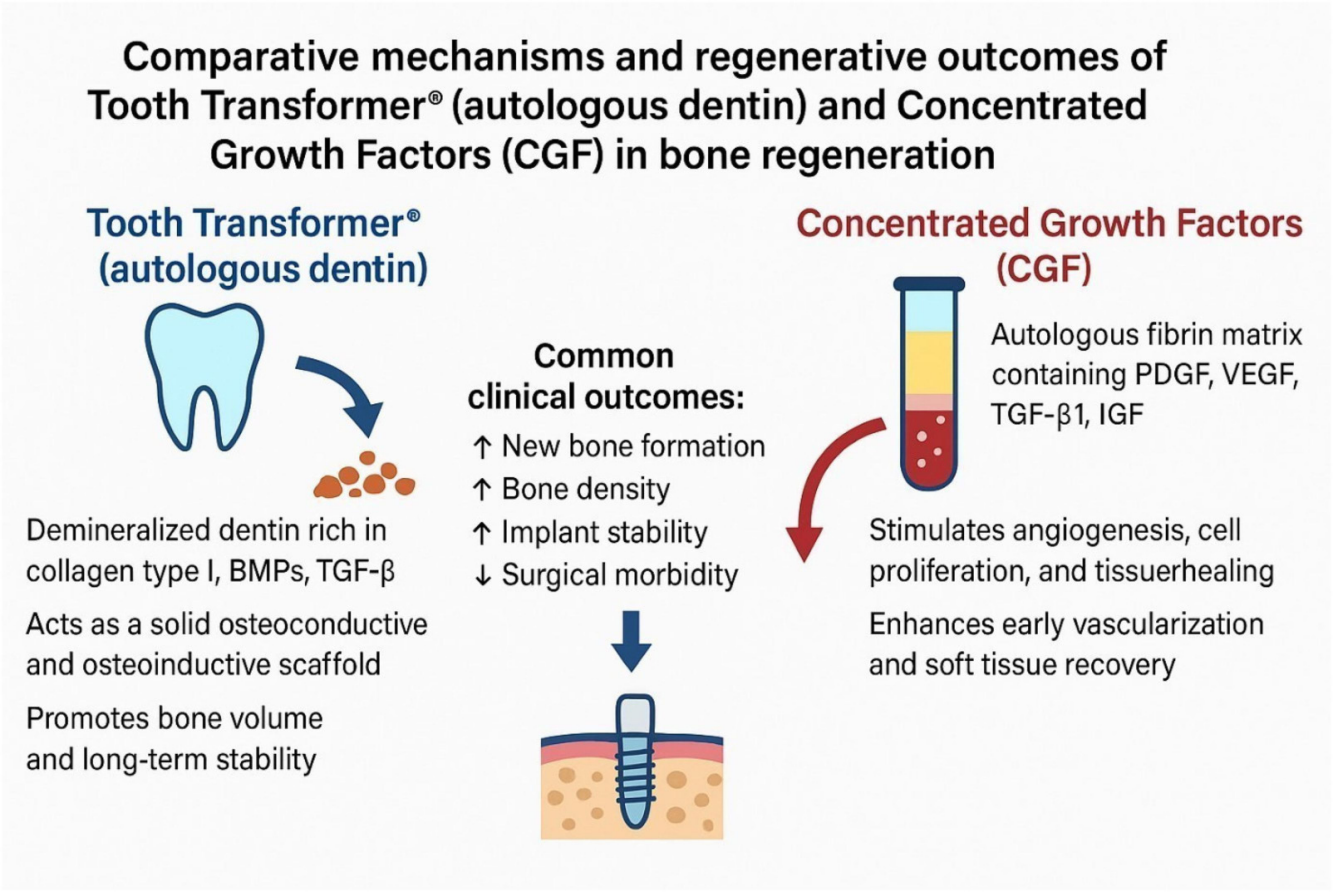
Comparative mechanisms of autologous dentin processed with the tooth transformer® and CGF in bone regeneration. Tooth- derived dentin provides an osteoconductive and osteoinductive scaffold releasing BMPs and TGF-β to promote stable bone formation, whereas CGF forms a fibrin matrix rich in PDGF, VEGF, and IGF that enhances angiogenesis and early tissue healing. Both biomaterials show high biocompatibility and complementary regenerative effects.

The comparative biological mechanisms and clinical effects of autologous dentin processed with the Tooth Transformer® and CGF are summarized in Figure 2.

In light of these considerations, the present systematic review aims to critically evaluate and compare the available clinical, radiographic, and histologic evidence concerning autologous dentin biomaterials processed with the Tooth Transformer® device and CGF obtained using autologous blood centrifugation systems. The specific objectives are: to assess the efficacy of each approach in achieving bone volume gain, bone density, and new bone formation; to analyze clinical and radiographic outcomes in different surgical indications (ridge preservation, sinus lift, cystic defect repair, ridge augmentation); to evaluate secondary outcomes including healing time, implant survival, and postoperative complications and to identify knowledge gaps and potential directions for future clinical research. By systematically comparing these two biologically distinct yet autologous regenerative methods, this review seeks to determine whether dentin-derived biomaterials or growth factor–based matrices provide superior or complementary benefits in bone regeneration. The findings are expected to contribute to the development of evidence-based, minimally invasive, and patient-specific regenerative protocols in oral and maxillofacial surgery.

## 2 Materials and methods

### 2.1 Study design and objective

This review was conducted according to PRISMA 2020 guidelines. In the present study, a *parallel narrative approach* was adopted, referring to an indirect qualitative comparison of two parallel bodies of evidence—autologous dentin grafts and autologous platelet concentrates—in the absence of head-to-head comparative clinical trials.

The main objective was to compare the clinical, radiographic, and histological outcomes of autologous dentin grafts processed with the Tooth Transformer® device (Tooth Transformer SRL, Milan, Italy) and CGF (autologous platelet concentrates, CGF, PRF, L-PRF, i-PRF, TPRF) in oral and maxillofacial bone regeneration procedures. The clinical performance and potential complementary roles of these autologous regenerative strategies in terms of bone formation, density, and clinical success, while minimizing surgical morbidity.

### 2.2 Research question (PICO)

Because CGF-only studies were limited, the PICO was updated as follows:

Population (P): Patients undergoing oral/maxillofacial bone regeneration (ridge preservation, sinus lift, cystic defect repair, ridge augmentation)
Intervention (I): Autologous dentin grafts processed with the Tooth Transformer®.
Comparison (C): Autologous platelet concentrates, including CGF, PRF, L-PRF, i-PRF, and TPRF.
Outcomes (O): Bone volume gain, radiographic density, histologic new bone formation; secondary outcomes: healing time, implant survival, complications.

The minimum follow-up period (T) required for inclusion was six months. The corresponding PICO research question was: *In patients requiring oral or maxillofacial bone regeneration, does the use of autologous dentin processed with the Tooth Transformer® lead to greater bone formation, density and clinical success compared to autologous platelet concentrates (CGF, PRF, L-PRF, i-PRF, TPRF), at a follow-up of at least six months?*

Due to the limited availability of CGF-only clinical studies, the comparison was extended to include the broader category of autologous platelet concentrates.

### 2.3 Search strategy

A comprehensive literature search was conducted across PubMed, Scopus, and Web of Science. The strategy combined Medical Subject Headings (MeSH) and free-text terms related to “autologous dentin”, “dentin graft”, “Tooth Transformer®”, “platelet concentrates”, “PRF”, and “CGF”. Boolean operators, field tags, and controlled vocabulary were adapted for each database to maximize retrieval sensitivity and specificity were iteratively refined based on preliminary results, and the final strategy was optimized to capture all relevant clinical studies involving autologous dentin grafts and autologous platelet-derived biomaterials in oral and maxillofacial bone regeneration. Restrictions regarding publication year were applied, and filters for human studies and English language were implemented where available. Studies were excluded for the following reasons: (1) the topic was not directly focused on autologous dentin or autologous platelet concentrates; (2) the design consisted of *in-vitro* or animal experiments; (3) the study reported fewer than five clinical cases; (4) Tooth Transformer® and CGF were applied in combination without separate analytical outcomes; (5) the article lacked quantitative bone-related results; or (6) the record corresponded to a review, editorial, commentary, or conference abstract without accessible full text. All search results were exported into a reference management software to remove duplicates and to ensure a standardized screening process. Titles and abstracts were independently assessed by two reviewers, followed by full-text evaluation according to predefined inclusion and exclusion criteria.

Last search date: December 30, 2025.

Filters: Humans, clinical studies, English language. Reference lists of included studies were hand-searched.

The complete search strategies for each database are reported in Table 1.

**Table 1:** Search strategy for 3 databases.

Database	Search strategy	Filters/limits
PubMed	[(“bone regeneration” OR “bone augmentation” OR “alveolar ridge” OR “ridge preservation” OR “sinus lift” OR “sinus floor elevation” OR “cystic defect”) AND (“Tooth Transformer” OR “autologous dentin” OR “dentin graft” OR “demineralized dentin”)] OR [(“bone regeneration” OR “bone augmentation” OR “alveolar ridge” OR “ridge preservation” OR “sinus lift” OR “sinus floor elevation” OR “cystic defect”) AND (“concentrated growth factors” OR CGF OR “platelet-rich fibrin” OR PRF OR “leukocyte-platelet rich fibrin” OR L-PRF OR “injectable PRF” OR i-PRF OR TPRF) AND (“oral surgery” OR “dental implant” OR “implant dentistry” OR “maxillofacial surgery”))	Humans; English; clinical studies; 2010–2025
Scopus	[TITLE-ABS-KEY(“bone regeneration” OR “bone augmentation” OR “alveolar ridge” OR “ridge preservation” OR “sinus lift” OR “sinus floor elevation” OR “cystic defect”) AND TITLE-ABS-KEY(“Tooth Transformer” OR “autologous dentin” OR “dentin graft” OR “demineralized dentin”)) OR [TITLE-ABS-KEY(“bone regeneration” OR “bone augmentation” OR “alveolar ridge” OR “ridge preservation” OR “sinus lift” OR “sinus floor elevation” OR “cystic defect”) AND TITLE-ABS-KEY(“concentrated growth factors” OR CGF OR “platelet-rich fibrin” OR PRF OR “leukocyte-platelet rich fibrin” OR L-PRF OR “injectable PRF” OR i-PRF OR TPRF) AND TITLE-ABS-KEY(“oral surgery” OR “dental implant” OR “maxillofacial surgery”))	English; 2010–2025; Humans; clinical studies
Web of Science	[TS = (“bone regeneration” OR “bone augmentation” OR “alveolar ridge” OR “ridge preservation” OR “sinus lift” OR “sinus floor elevation” OR “cystic defect”) AND TS = (“Tooth Transformer” OR “autologous dentin” OR “dentin graft” OR “demineralized dentin”)) OR [TS = (“bone regeneration” OR “bone augmentation” OR “alveolar ridge” OR “ridge preservation” OR “sinus lift” OR “sinus floor elevation” OR “cystic defect”) AND TS = (“concentrated growth factors” OR CGF OR “platelet-rich fibrin” OR PRF OR “leukocyte-platelet rich fibrin” OR L-PRF OR “injectable PRF” OR i-PRF OR TPRF) AND TS = (“oral surgery” OR “dental implant” OR “maxillofacial surgery”))	English; 2010–2025; clinical studies; Humans.

### 2.4 Eligibility criteria

Inclusion criteria:

- Human clinical studies (RCTs, prospective/retrospective trials, case series ≥5 patients)
- Use of TT or platelet concentrates as the primary regenerative biomaterial
- Reporting clinical, radiographic or histological bone outcomes
- English language (2010–2025)

Exclusion criteria:

- Animal or *in vitro* studies
- Case reports with <5 patients
- Reviews, editorials or abstracts without full data
- Studies using TT + CGF without separate data analysis

### 2.5 Data extraction and analysis

Data extraction was performed independently by two reviewers using a standardized Microsoft Excel sheet designed for systematic reviews. The following information was collected for each included articles: author and year of publication, country, study design, sample size, anatomical site, type of intervention (Tooth Transformer® or CGF), follow-up duration, main outcomes, quantitative results, complications, and authors' conclusions.

### 2.6 Quality assessment

The methodological quality and risk of bias of the included studies were independently evaluated by two reviewers.

For randomized controlled trials, the Cochrane Risk of Bias 2 (RoB 2) tool was applied; for non-randomized studies, the MINORS (Methodological Index for Non-Randomized Studies) criteria were used; and for case series, the JBI Critical Appraisal Checklist was employed. Any discrepancies were resolved through discussion. Overall, the included studies demonstrated moderate to high methodological quality, although variability in sample size, outcome measures and follow-up duration were observed across trials. A comprehensive risk-of-bias table (RoB2, MINORS, JBI) has been included as Table 2.

**Table 2:** Overview of the studies discussed in the present review.

Author (Year)	Country	Study design	Sample size	Anatomical site	Intervention	Follow-up	Main outcomes	Quantitative results	Complications	Authors’ conclusions
Talaat et al. (2018)	Egypt	Prospective clinical study	20	Mandibular cystic defects	BMC + CGF	12 months	Bone regeneration	Higher bone density vs control (CBCT)	None reported	Safe and effective autologous approach
Minetti et al. (2023)	Italy	Experimental + radiographic clinical study	NR	Regenerated bone sites	Tooth Transformer® dentin	NR	Granule morphology, handling	Optimal porosity and stability	None reported	Suitable autologous dentin graft
Minetti et al. (2025)	Italy	Long-term clinical follow-up	NR	GBR implant sites	Tooth Transformer® dentin	6 years	Implant survival, bone stability	97% implant survival; >90% volume maintenance	None reported	Long-term reliability confirmed
Dipalma et al. (2025)	Italy	Retrospective clinical study	31	Maxillary atrophy	TT dentin + CGF	84 months	Bone regeneration, implant success	Stable 3D bone regeneration	None reported	Synergistic effect of dentin + CGF
Medeiros-Monzón et al. (2025)	Spain	Prospective clinical study	NR	MRONJ sites	L-PRF	NR	Healing, recurrence	Improved mucosal healing	Reduced recurrence	Regenerative and anti-inflammatory effect
Hashmi et al. (2025)	India	Comparative clinical study	NR	Third-molar sockets	PRF + xenograft	NR	Bone fill, density	Superior bone fill vs control	None reported	Enhanced regeneration with PRF
Gupta et al. (2025)	India	Double-blind RCT	NR	Maxillomandibular defects	i-PRF + HA	NR	Bone density, healing	+25% bone density vs HA	None reported	Synergistic regenerative effect
Martande et al. (2016)	India	Randomized controlled trial	60	Periodontal intrabony defects	PRF + atorvastatin	9 months	PD reduction, bone fill	Significant bone fill increase	None reported	Additive regenerative benefit
Andrade et al. (2020)	Brazil	Pilot clinical study	NR	Post-extraction sockets	Dentin + L-PRF	NR	Ridge preservation	Better volume maintenance	None reported	Effective autologous matrix
Chatterjee et al. (2016)	India	Randomized comparative study	38	Periodontal intrabony defects	PRF/TPRF	9 months	PD reduction, CAL gain	Significant improvement vs OFD	None reported	Comparable regenerative efficacy

### 2.7 Ethical considerations

As this review analyzed data derived exclusively from previously published studies, ethical approval was not required. The study protocol adhered strictly to the PRISMA 2020 guidelines. The review was not prospectively registered in PROSPERO at the time of submission, was registered in the International Prospective Register of Systematic Reviews (PROSPERO) under the registration number [1185676].

## 3 Results

**Figure 3:**
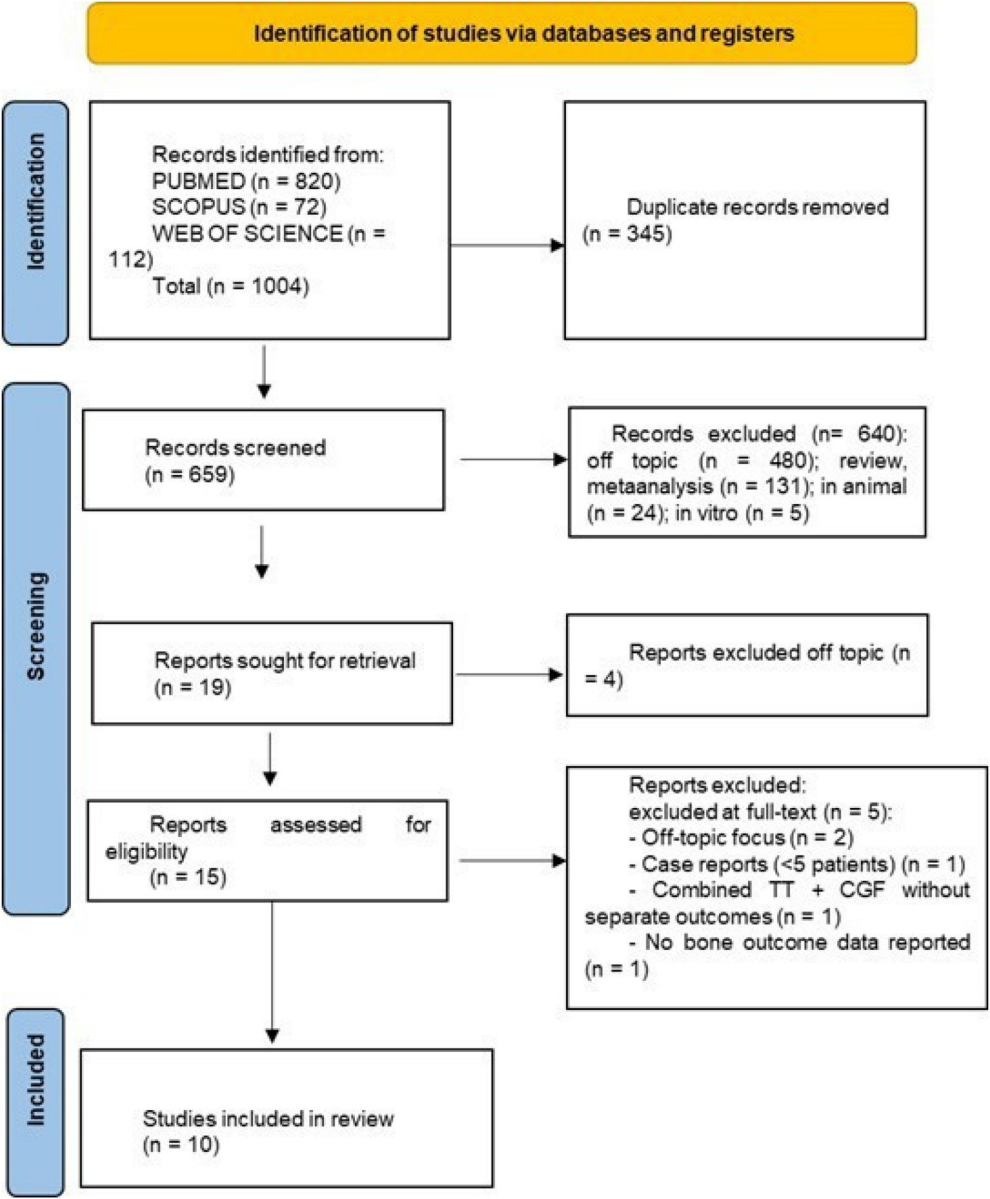
Prisma flow chart.

A total of 1,004 records were identified through electronic database searches (PubMed, Scopus and Web of Science) and manual screening of relevant bibliographies. After the removal of 345 duplicate records, 659 unique articles were assessed by title and abstract. Following the application of the predefined inclusion and exclusion criteria, 640 studies were excluded for irrelevance, off-topic focus, review or meta-analysis nature, or use of animal or *in vitro* models. Consequently, 19 full-text studies were initially included in the qualitative synthesis. However, after a final eligibility assessment, only 10 studies met all inclusion criteria and were analyzed in detail and discussed within this review. The study selection process is summarized in Figure 3 (PRISMA 2020 flow diagram), while the main characteristics and outcomes of the included studies are presented in Table 2.

### 3.1 Quality and risk of bias assessment for the included articles

Among the ten included studies, the domain most frequently associated with a high risk of bias was the randomization process. This was primarily due to the inclusion of retrospective studies, pilot trials, and prospective designs without random allocation. In contrast, the domains related to missing outcome data and outcome measurement generally showed a low risk of bias, as most studies provided adequate follow-up and employed standardized radiographic or clinical evaluation methods. The assessment of deviations from intended interventions revealed some concerns in studies where blinding or protocol adherence was not clearly described. Selective reporting was occasionally rated as having some concerns, particularly in non-randomized studies that did not register a protocol or failed to report all predefined outcomes. Overall, randomized controlled trials demonstrated higher methodological rigor, while non-randomized designs, although informative, exhibited greater variability and limitations that must be considered when interpreting the findings of this systematic review. Risk of bias was independently assessed by two reviewers using RoB 2 for randomized trials, MINORS for non-randomized studies, and the JBI checklist for case series.

Disagreements were resolved by consensus. Results are summarized in Table 3.

**Table 3:** Risk of bias summary for included studies.

Study (author, year)	Randomization	Deviations from interventions	Missing data	Outcome measurement	Selective reporting
Talaat et al. (2018)	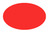	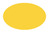	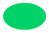	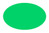	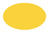
Minetti et al. (2023)	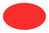	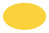	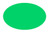	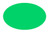	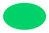
Minetti et al. (2025)	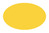	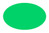	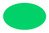	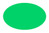	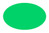
Dipalma et al. (2025)	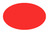	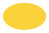	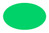	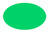	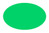
Medeiros-Monzón et al. (2025)	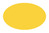	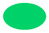	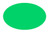	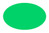	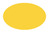
Hashmi et al. (2025)	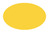	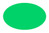	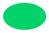	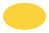	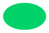
Gupta et al. (2025)	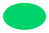	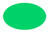	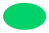	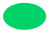	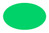
Martande et al. (2016)	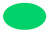	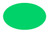	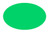	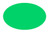	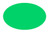
Andrade et al. (2020)	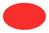	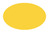	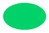	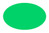	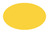
Chatterjee et al. (2016)	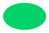	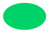	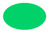	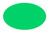	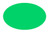

### 3.2 Biomaterials derived from the tooth transformer®

Studies focusing on the Tooth Transformer® (TT) highlight the crucial role of demineralized autologous dentin as both a structural and bioactive material for bone regeneration.

In particular, Minetti et al. performed a dimensional and morphological analysis of the granules produced with the TT® device, demonstrating that the microstructure of the material characterized by a microporous surface and a homogeneous distribution of hydroxyapatite and type I collagen promotes cellular migration and osteoblast adhesion. The study also showed that the chemical composition of the dentin graft closely resembles that of human cortical bone, supporting physiological bone regeneration with rapid remodeling and excellent vascular integration (238).

In a prospective six-year follow-up, Minetti et al. clinically confirmed the volumetric stability and predictability of TT in guided bone regeneration using autologous dentin grafts. Implants placed in treated sites showed a 97% survival rate, with an average volumetric maintenance exceeding 90% and excellent radiographic bone density. The authors concluded that standardized processed demineralized dentin demonstrated clinically stable outcomes over time; however, direct comparative trials against heterologous or synthetic materials are currently lacking, and therefore no definitive claim regarding replacement can be made (207).

Further evidence was provided by Dipalma et al. who retrospectively evaluated the clinical outcomes at 84 months of patients with maxillary atrophy treated with Tooth Transformer® and Concentrated Growth Factors (CGF). The combination of the two biomaterials achieved stable three-dimensional bone regeneration, with a significant increase in trabecular density and complete replacement of residual dentin with vital bone. The combined use of TT and CGF proved particularly effective for regenerating large defects, reducing healing time and improving the histological quality of newly formed bone (239).

The combination approach was also investigated by Andrade et al. who tested a matrix composed of autologous dentin particles, L-PRF, and fibrinogen for alveolar ridge preservation. The experimental group showed better maintenance of the alveolar profile and more uniform bone formation compared to controls. The authors emphasized that the synergy between the mineral component of TT and the bioactivity of fibrin enables faster and more stable regeneration, confirming the efficacy of combining autologous biomaterials (240).

Overall, studies on the Tooth Transformer® confirm that demineralized autologous dentin possesses osteoconductive, osteoinductive, and resorbable properties, with specific positive outcomes for bone regeneration: three-dimensional gains useful for implant surgery, bone density compatible with mature bone, and documented long-term volumetric stability.

### 3.3 Autologous growth factor concentrates (CGF, PRF, i-PRF, TPRF)

Studies on CGF/PRF have shown that these blood-derived products are not mineral substitutes but rather stimulate healing, with a measurable impact on bone regeneration (bone fill, density, defect closure), particularly in contained or post-extraction defects.

Talaat et al. demonstrated that the combined application of autologous bone marrow concentrate and CGF in post-enucleation mandibular defects promotes rapid bone regeneration, with the formation of mature lamellar bone within six months. The combination reduced the risk of residual cavities and accelerated healing, highlighting the regenerative potential of concentrated growth factors within the fibrin matrix (241).

Gupta et al. conducted a double-blinded, randomized controlled clinical trial on patients with maxillomandibular bony defects, comparing the use of injectable PRF (i-PRF) polymer combined with hydroxyapatite vs. hydroxyapatite alone. The results showed an average 25% increase in bone density in the i-PRF group, along with significantly reduced postoperative healing time. The efficacy was attributed to the sustained release of angiogenic and osteopromotive cytokines from PRF (242).

Similarly, Martande et al. evaluated the combination of PRF and 1.2% atorvastatin for the treatment of intrabony periodontal defects. The study reported significant clinical attachment gain, reduced probing depth, and increased radiographic bone density. The authors hypothesized that atorvastatin, by stimulating BMP-2 expression (present in dentin derived from autologous teeth), enhances the regenerative action of PRF, providing dual osteogenic and angiogenic stimulation (243).

Hashmi et al. compared sticky bone (xenograft combined with PRF) with simvastatin application in post- extraction sockets. The PRF-treated group exhibited faster healing, better trabecular organization, and superior volumetric preservation compared to the control group, confirming PRF's role as a cohesive and bioactive matrix (244).

A significant clinical contribution was provided by Chatterjee et al. who compared PRF and titanium PRF (TPRF) for the treatment of intrabony periodontal defects vs. surgical debridement alone. Both materials produced significant improvements in clinical parameters, with TPRF showing greater stability and prolonged growth factor release, rendering it slightly more effective at nine months follow-up.

Finally, Medeiros-Monzón et al. conducted a prospective study on the use of L-PRF for the treatment and prevention of medication-related osteonecrosis of the jaw (MRONJ).

PRF application significantly reduced symptoms, improved mucosal and bone healing, and decreased the incidence of recurrence. Although three-dimensional bone gain was not measured, these results are highly relevant for regeneration: in biologically compromised bone, PRF restores pro-regenerative conditions (angiogenesis, inflammation modulation) that support bone maintenance and repair (245).

In summary, autologous platelet concentrates—including CGF, PRF, L-PRF, i-PRF, and TPRF—show a consistent capacity to enhance early healing and bone maturation. However, because most available studies investigate PRF-based formulations rather than CGF alone, the findings should be interpreted as reflective of the broader family of platelet concentrates. Moreover, their effects pertain primarily to biological stimulation rather than volumetric augmentation, and direct comparison with TT-derived dentin is not possible based on current evidence.

## 4 Discussion

This systematic review included ten clinical and experimental studies analyzing two autologous bone regeneration strategies dentin biomaterials processed with the Tooth Transformer® (TT) and growth factor concentrates (CGF, PRF, i-PRF, TPRF) (246, 247). In this review, the focus is not only on biocompatibility but also on bone-related outcomes, including bone gain (mm), radiographic density (HU), marginal stability, and implant survival rates. Autologous dentin provides an osteoconductive structure capable of maintaining space and supporting three-dimensional augmentation, whereas platelet concentrates stimulate bioinduction and angiogenesis, resulting in accelerated bone maturation and enabling rapid primary implant stability.

No randomized or controlled clinical trials directly comparing Tooth Transformer® dentin and CGF/PRF/i- PRF were identified in the literature. Therefore, all conclusions represent indirect qualitative comparisons. However, because no direct head-to-head comparative clinical trials between Tooth Transformer® grafts and platelet concentrates were identified, the present analysis represents an indirect qualitative comparison rather than a true assessment of superiority. The findings should therefore be interpreted with caution and within the limitations imposed by heterogeneous methodologies, clinical indications, and outcome measures across studies. Overall, both autologous strategies demonstrated favorable regenerative outcomes, although their roles appear biologically distinct: autologous dentin primarily provides a mineralized structural scaffold, while platelet concentrates act mainly as bioactive enhancers of healing and angiogenesis.

### 4.1 Comparative analysis and integrated perspective

The comparison between the two approaches highlights a clear functional and biological complementarity in bone regeneration. To date, no clinical study has directly compared autologous dentin and CGF in a randomized or controlled design, limiting the strength of comparative conclusions. The Tooth Transformer® provides a stable structural support, with pronounced osteoconductive properties and a proven capacity to maintain volume over the long term, making it particularly suitable for sinus floor elevations and extensive bone defects where predictable three- dimensional augmentation is required.

In contrast, autologous growth factor concentrates, such as CGF, PRF, i-PRF, and TPRF, exert primarily a bioactive effect: they stimulate vascularization, modulate the inflammatory response, and accelerate the maturation of newly formed bone, enhancing the density and quality of the regenerated tissue, especially in contained defects, post-extraction sites, and intrabony periodontal defects.

As highlighted by Dipalma et al., the combination of the two materials: may offer synergistic benefits in selected clinical scenarios the use of autologous dentin processed with the Tooth Transformer® as a mechanical scaffold, together with the growth factor-rich fibrin matrix of CGF, allows for simultaneous optimization of vertical and volumetric bone gain, regenerated tissue density, and healing speed.

Long-term follow-up (up to 84 months) from Dipalma et al. reported stable volumetric maintenance and absence of immunologic reactions or graft-related complications.

However, because the available evidence is limited to retrospective and heterogeneous clinical studies, these observations should be interpreted cautiously and cannot be considered conclusive regarding superior performance or universal applicability.

At the cellular level, demineralized dentin releases BMP-2, TGF-β and osteopontin, promoting osteoblast differentiation, while CGF provides VEGF- and PDGF-rich fibrin matrices that accelerate angiogenesis. Their combination may theoretically create a synergistic environment supporting both scaffold stability and biologically enhanced healing.

Based on the available evidence, autologous dentin appears particularly suitable for defects requiring space maintenance (sinus lift, vertical augmentation), whereas CGF/PRF is more appropriate in contained or small defects, postoperative sockets, and compromised healing sites. Combined use may be beneficial in large defects requiring both volume stability and enhanced angiogenesis.

The main limitations common to the analyzed studies include methodological variability, small sample sizes, the use of different measurement scales (millimeters, percentage of bone fill, Hounsfield Units), and the lack of standardized protocols for histologic and radiographic evaluation. Nevertheless, the available evidence supports the potential clinical value of both autologous approaches, although the absence of direct comparative trials prevents establishing relative effectiveness. Their integration appears biologically plausible, but further controlled research is required before recommending standardized combined protocols.

### 4.2 Final considerations and conclusions

This systematic review indicates that autologous dentin processed with the Tooth Transformer® and autologous platelet concentrates (CGF/PRF/L-PRF/i-PRF/TPRF) are both safe and clinically effective adjuncts for oral and maxillofacial bone regeneration, but they act through complementary mechanisms and therefore address different clinical needs. Autologous dentin primarily behaves as a mineralized scaffold with space-maintaining capability and favorable volumetric stability, supporting three-dimensional augmentation and providing a substrate that is compatible with mature bone density over time. In contrast, platelet concentrates mainly enhance the biological phase of healing by promoting angiogenesis, modulating inflammation, and accelerating early tissue maturation, with particularly consistent benefits in contained defects and post-extraction or intrabony sites where rapid soft- and hard-tissue healing is clinically advantageous. From a clinical perspective, these findings support a pragmatic indication-based approach: dentin grafts appear more suitable when defect morphology requires reliable space maintenance and long-term volume preservation (e.g., larger or non-contained defects, ridge augmentation, sinus floor elevation), whereas platelet concentrates may be preferentially considered when the primary goal is to optimize early healing and regenerative dynamics (e.g., contained defects, periodontal intrabony defects, extraction sites, compromised soft-tissue conditions). Importantly, the available evidence suggests that combined protocols may be particularly promising, as they can couple structural stability (dentin) with biologic stimulation (platelet concentrates), potentially improving handling, accelerating healing, and enhancing the quality of regenerated tissue. However, the current body of literature does not include direct head-to-head comparative clinical trials and remains heterogeneous in study design, indications, outcome measures, and follow-up durations, which prevents quantitative synthesis and limits the strength of comparative inferences. Future well-designed, multicenter randomized controlled trials with standardized endpoints (linear/volumetric bone gain, HU-based density, histologic new bone percentage, implant survival, marginal bone stability, and complication rates) are needed to define clear clinical algorithms and to validate the long-term efficacy of combined autologous approaches.

At the same time, autologous growth factor concentrates (CGF, PRF, i-PRF, TPRF) are confirmed as highly effective biological tools for enhancing bone regeneration: they accelerate healing, promote angiogenesis, improve trabecular organization and the quality of newly formed tissue, and reduce the risk of postoperative complications, especially in more contained defects or sites with compromised healing.

The combination of the two approaches, employing an autologous structural scaffold (TT dentin) together with a bioactive fibrin matrix (CGF/PRF), emerges as the most promising solution to achieve an optimal balance between the quantity and quality of regenerated bone, long-term stability, and reduced healing time.

The combination of the two approaches employing an autologous structural scaffold (TT dentin) together with a bioactive fibrin matrix (CGF/PRF) may represent a promising strategy to enhance both structural stability and biological stimulation. However, current evidence is limited and heterogeneous, and further comparative clinical trials are necessary before definitive conclusions or routine clinical recommendations can be established.

That clarify the specific indications, advantages, and limitations of autologous dentin, platelet concentrates, and their combined use in regenerative oral surgery.
